# Chemical roots of biological evolution: the origins of life as a process of development of autonomous functional systems

**DOI:** 10.1098/rsob.170050

**Published:** 2017-04-26

**Authors:** Kepa Ruiz-Mirazo, Carlos Briones, Andrés de la Escosura

**Affiliations:** 1 Biofisika Institute (CSIC, UPV/EHU), 48940 Leioa, Spain; 2 Department of Logic and Philosophy of Science, University of the Basque Country, 20018 Donostia – San Sebastián, Spain; 3 Department of Molecular Evolution, Centro de Astrobiología (CSIC–INTA, Associated to NASA Astrobiology Institute), 28850 Torrejón de Ardoz, Madrid, Spain; 4 Organic Chemistry Department, Universidad Autónoma de Madrid, 28049 Cantoblanco, Madrid, Spain; 5 Institute for Advanced Research in Chemical Sciences (IAdChem), 28049 Cantoblanco, Madrid, Spain

**Keywords:** chemical evolution, natural selection, origins of life, autonomous functional systems, prebiotic systems chemistry

## Abstract

In recent years, an extension of the Darwinian framework is being considered for the study of prebiotic chemical evolution, shifting the attention from homogeneous populations of naked molecular species to populations of heterogeneous, compartmentalized and functionally integrated assemblies of molecules. Several implications of this shift of perspective are analysed in this critical review, both in terms of the individual units, which require an adequate characterization as self-maintaining systems with an internal organization, and also in relation to their collective and long-term evolutionary dynamics, based on competition, collaboration and selection processes among those complex individuals. On these lines, a concrete proposal for the set of molecular control mechanisms that must be coupled to bring about autonomous functional systems, at the interface between chemistry and biology, is provided.

## 1. Introduction

About a century and a half ago, in his book *On the Origin of Species*, Charles R. Darwin proposed natural selection (NS) as the main driving force that guides the evolution of species, conceived as a process of descent with modification from a common ancestor. Today we have overwhelming evidence to support that this process has been continuously shaping the living world over time on the Earth and, thus, it constitutes a central paradigm of biology. Darwinian evolution must have started, at the latest, with the first population of living cells, whose precise characteristics remain inaccessible to us, but whose descendants originated, among other putative lineages later extinct, the so-called *last universal common ancestor* (LUCA) [1]. Without excluding other important factors (e.g. genetic drift, gene flow, population size/structure effects, symbiotic and mutualistic relationships, lateral gene transfer and sex), past and current biodiversity is therefore regarded as the outcome of evolutionary dynamics based on NS from LUCA [2].

Nevertheless, a big scientific riddle not yet resolved is what happened before the first self-reproducing cellular organisms appeared on Earth, likely between 3.8 and 3.5 billion years ago. Although biological systems consist of well-described sets of coupled chemical reactions, very little is known about the transition from inanimate matter to living entities, i.e. from the most complicated chemistry we can think of to the simplest biology. The majority of researchers in the field of origins of life have worked under the assumption that a population of self-replicating RNA (or RNA-like) molecules started competing for a limited amount of resources (i.e. nucleotides or analogous monomers) in their local environment, and some form of NS already began operating at that chemical level [3–5]. This widespread conception is supported by two breakthroughs from last half-century molecular biology: (i) the development of *in vitro* molecular evolution technologies, which allowed to prove that populations of biopolymers with template properties could, indeed, change in time under artificial selective pressure [6–9]; and (ii) the discovery of *ribozymes* [10,11], which demonstrated the catalytic capacities of RNAs and, thereby, the dual role that this type of macromolecule might have played at the onset of life.

As a result, some researchers have conjectured on a possible link between the emerging concept of *chemical evolution* and the established notion of *biological evolution*, considered as two different stages of a common natural process of matter complexification [12–15]. Uncovering possible mechanisms of chemical evolution that include early or minimalist versions of NS would then be crucial to understand how life originated on Earth—or, eventually, in a different extraterrestrial environment. Computational and theoretical studies carried out in the last years support that *chemical kinetics* may become *evolutionary dynamics* in populations of self-replicating molecules, or in collectively autocatalytic networks [16–18]. The overall idea behind these studies is that the evolutionary potential of such chemical systems would result from an increase in their dynamic stability as a consequence of the copying process or the cross-catalytic effects among the molecules in the network.

Several key questions remain open in this context, however. First, why is it so hard to find artificial chemistries that spontaneously converge into systems or phenomena of higher complexity and dynamic stability? Is it because experimental scientists are not finding the right precursors and boundary conditions? Or is it because there are ‘evolutionary bottlenecks’—still to be identified and characterized—that must be overcome to trigger off such a behaviour? Secondly, is ‘kinetic control’ (i.e. the rate of template copying or the catalytic power of the molecules) the only relevant aspect to ensure the necessary dynamic robustness of those prebiotic systems that would, eventually, lead to the onset of open-ended, biological evolution? If not, what other types of control mechanisms or basic functions should be taken into account at those initial stages? More precisely, regarding the emergence of Darwinian evolutionary dynamics, how large must a primary ‘functional/phenotypic space’ be for NS to get established, or become operational? And, finally, granted that cyclic chemical reaction networks are so important for the living, how could they be initially kept together, away from thermodynamic equilibrium for long enough, so that they may actually reproduce and become evolvable individuals?

**Figure 1.**
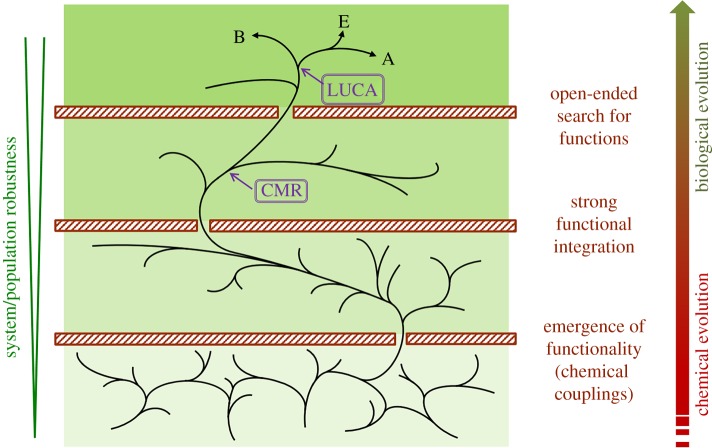
Scheme of the different stages and bottlenecks that could have occurred during the transition from chemical to biological evolution. Among the complex and interacting chemical mixtures present on early Earth, only those that developed the first functional couplings were available for the next step of the overall process. Further diversification of those coupled systems made stronger functional integration mechanisms possible, leading to—at least—one type of system in the population with sufficient dynamic/structural stability to overcome the bottleneck imposed by the need for reliable reproduction (including molecular template mechanisms). This stage corresponded to systems in which compartment, metabolism and replication (CMR) were tightly coupled. In turn, only those CMR systems that became capable of open-ended biological evolution (see explanation in the text) would show the long-term robustness required to follow the pathway to LUCA and its further diversification into the three domains of life.

Questions like the previous ones were rarely asked in the field of origins of life 20 years ago, when different research schools were still fighting for the prebiotic plausibility of their preferred biopolymer (e.g. nucleic acid versus protein) or biochemical process (template replication versus metabolic pathways). However, after the advent of *systems* approaches in biology at the turn of the century [19–21], chemistry is also taking up the challenge of dealing with increasingly intricate combinations of molecules at once, under various conditions and making use of novel experimental set-ups and technologies [22–27], which opens new perspectives and opportunities for research on the origins of life [28–31]. In that context, figure 1 introduces our view about the main stages and bottlenecks involved in the transition from chemical to biological evolution, with emphasis on the evolutionary importance of establishing efficient functional couplings within chemical systems, as we will argue in more detail below. Of course, chemical evolutionary pathways (bottom part of the figure) will surely cover a wider research area than those potentially conducive to prebiotic phenomena. Nevertheless, some of them should turn into legitimate candidates to bring about lifelike dynamic behaviours, perhaps by means of alternative components and processes to the ones actually implemented in our biosphere. Despite the expected difficulties in establishing the ‘liveliness’ of these intermediate chemical systems, the necessity to start integrating *organizational* and *evolutionary* accounts of those kinds of complex (pre-bio)molecular phenomena is becoming less and less controversial, and that is precisely the area on which we focus this short critical review or perspective article.

## 2. Merging systems chemistry and evolutionary theory

It is turning increasingly apparent that evolutionary theory can no longer keep the ‘black box’ of individuality closed. In particular, the triad ‘multiplication, variation and heredity’ cannot be simply assumed as a general set of conditions that define any *unit of selection* [2], but needs to be more precisely specified, if it is to carry any descriptive/predictive power. In turn, those approaches that invoke physico-chemical forces (e.g. self-organization and self-assembly, spatial constraints and energetic couplings) to support mechanistic or physiological accounts for the development of chemical complexity on the way to biological phenomena should be made coherent with the Darwinian framework, which explains the dynamics taking place at a population level. Oversimplifications, imposing either the evolutionary framework (i.e. disregarding the internal complexity of the individuals) or the physiological/organizational one (i.e. disregarding the power of evolutionary mechanisms), have not worked satisfactorily [32,33]. Thus, the challenge for origins-of-life research is to make use of systems chemistry to identify and characterize mixtures of biomolecular precursors that could get coupled into individuals with capacity for self-maintenance and potential increase in complexity (i.e. ‘proto-organisms’, taken as assemblies of different chemical species with functional attributes—see below). In parallel, researchers should carefully examine the evolutionary consequences of having one type of individual or another in the population, in terms of the competitive and selective dynamics that take place at that collective level, within a plausible geochemical setting.

Two additional arguments can be given to support the necessity of merging the new *systems* approach and evolutionary theory. First, all known living beings are made of highly diverse and intertwined components and transformation processes. Simplifying their origins to the dynamics of one type of molecular component is a strong and reductionist conjecture, which should not be taken for granted. Second, in order to turn operational and lead to systems of increasing complexity, evolution by NS should have required, from the beginning, individuals who provide a wide enough space to express a phenotypic variety [34,35]. For example, although molecular evolution through artificial selection for a given phenotype (e.g. a given aptamer or an engineered ribozyme) has been clearly demonstrated to take place without any need for such ‘composite individuals’ [6–9,36], it is becoming more and more obvious that molecules of a single kind, no matter their structural complexity, face intrinsic bottlenecks due to the fact that they do not offer the wide phenotypic space required for the emergence of autonomous functional systems. The alternative scenario here proposed is not straightforward, either. In particular, it requires a high degree of chemical diversity and heterogeneity from the beginning, an apparent complication that some other researchers have also supported, with different strength and motivations [37–40]. However, this is bound to pay back soon in terms of the behavioural richness and evolutionary potential displayed by the more complex individuals that are drawn in the process. Somehow (as we explain in more detail in the sections below), reaching that initial threshold of complexity would make it possible, for chemical systems that overcome a first set of evolutionary bottlenecks (bottom part of figure 1) and keep relatively robust dynamics, to display a wide range of chemical couplings and functional behaviours that would allow them, in turn, to undergo further prebiotic transitions (in particular, to originate ternary systems denoted as ‘CMR’ in figure 1).

On these lines, acknowledging the importance of the input provided by the *RNA World* theory (as it was proposed 30 years ago [41], but also, more interestingly, through the recent advances in the prebiotic synthesis of its precursor molecules [42–45] or the improved elaborations on the general hypotheses behind it—both theoretical [46–48] and experimental [49,50]), one should nevertheless accept that an important gap remains in our understanding of the processes that could have spontaneously supported the emergence, maintenance and evolutionary potential of such a precellular world. Especially, it must be noted that various chemical reactions traditionally involved in the artificial organic syntheses of RNA precursors are thermodynamically uphill or show high kinetic barriers in the absence of activating molecules or catalysts, respectively. It looks highly more probable that before (or at the same time as) biopolymers with functional capacities came to the stage, other (simpler) molecular species and structures (e.g. small, non-coded peptides) had to be present, in close interaction with them. This is so because the latter would have provided, precisely, the *enabling conditions* for the robust, autonomous production of the former, as well as the *phenotypic context* within which those emergent biopolymers could carry out and unfold their diverse functional roles, including template replication.

The problem is intricate, though: approaches that have suggested self-sustaining autocatalytic networks as an alternative basis for the origins of life (i.e. metabolism-first scenarios [51–56]) also face their own set of problems. Potentially capable of robust self-maintenance, autocatalytic networks have nevertheless obtained very little experimental support in prebiotic conditions [57,58], and hold severe theoretical limitations as well (e.g. the depletion of certain network intermediates when side reactions are considered, or the lack of evolvability in the absence of some mechanism of heredity [16]).

In addition, both ‘replication-first’ and ‘metabolism-first’ approaches face further trouble, such as the permanent threat of dilution and the spatio-temporal coordination of the underlying processes. These difficulties seem to force the inclusion of self-assembled compartments in the picture, as they incorporate selectively permeable boundaries that could keep concentration levels of the relevant chemical species above critical thresholds, and would mark a clear ‘inside–outside’ distinction (i.e. a strong criterion of individuality). At the same time, the presence of such compartments (commonly assumed to be vesicles with an internal aqueous core, but perhaps initially reducible to simpler, two-phase systems—like droplets or Oparin's ‘coacervates’) would provide the adequate scale and scaffold for molecules to start getting organized functionally and would turn those complex molecular systems into lifelike individuals with capacity to evolve as protocellular systems (e.g. [59,60]).

Thus, one can reformulate the question of the origins of life in the following global terms: before full-fledged biological systems appeared on Earth, what systems could overcome the apparent thermodynamic barrier to complexification and produce the rather intricate self-reproducing entities that initiated evolution in a Darwinian sense? There is no current answer for this riddle; therefore, we need a more general conceptual framework to understand chemical evolution. The required theory should account for the increase in complexity that occurred in prebiotic times, when chemistry was already complex and heterogeneous but still far from generating the self-replicating polymers, the self-sustaining protometabolic cycles and the self-multiplying protocellular compartments whose efficient functional integration brought about the first living systems. In the remaining sections of this prospective review, a first sketch of what such a new conceptual framework might look like is offered, relating it to several key advances that have occurred in the field of ‘prebiotic systems chemistry’ [28] during the last few years.

## 3. Chemical evolution through the development of autonomous functional systems

Our perspective considers an extension of the Darwinian framework for the study of prebiotic chemical evolution, but not in the traditional way that this has been carried out within the field of origins (i.e. searching for minimal self-replicating molecular systems [61–65]) or, more recently, with the inclusion of compartments under a similar scheme [60,66–68]. Instead, we suggest to apply an evolutionary account in the context of *autonomous* systems that are made of diverse combinations of chemical precursors (i.e. (bio)molecules and their supramolecular assemblies). The underlying hypothesis is that, under adequate physico-chemical conditions, those molecular species and assemblies will achieve a collective reinforcement through various types of interaction (e.g. mutual chemical transformations, recognition and control relationships, self-assembly, pattern formation, collective synchronization phenomena and physical boundary effects). Consequently, some of these mixed bio-precursors, together with the processes that they trigger off in interaction, will increase their dynamic/structural stability by becoming functionally engaged with each other. This should enable researchers to design experiments of competition and selection among different types of such individuals, in order to test their actual dynamic robustness and evolutionary potential.

Central to our account is the idea of *function*, a key concept in biology that is projectable to a chemical scenario. By function we understand any distinct contribution, by a distinguishable part of a system, to the maintenance of that system as a whole [69,70]. In other words, we take a dispositional (organizational) standpoint with regard to this concept, which highlights its *relational* character. A part of the system (e.g. a molecule), by itself, does not have a function. It can only have it in connection to other components of such a system, that is, in the context of an *organization*. In general, an organization can belong to the natural world or be human-made (e.g. an artificial device or machine). The main difference between them consists in whether the function is defined externally (i.e. by the person building the machine, with all its parts) or has an endogenous source and goal. We are interested in spontaneously formed, autonomous systems, like living organisms, so the focus here will be on the latter.

A preliminary reflection, from the definition just given, is that it will be hard—if not impossible—to find natural systems with only one or two functions. Accordingly, one of the key issues to address is determining the minimal number and type of chemical components/interactions required to obtain a system in which relationships can start being properly considered as *functional*. In other words, a central motivation behind our proposal consists in naturalizing an organizational conception of function, endowed with biological (i.e. physiological) meaning, but well-grounded in *evolutionary systems chemistry*.

This perspective may hold some similarities with other theoretical schemes recently developed with the aim to explain the evolution of complex systems. Pascal *et al.* [14], for example, argue about a sequence of stages in which chemical systems of increasing stability or robustness appear, thus opening the possibility for new developments within the time window allowed by their relative stability. In addition to some thermodynamic considerations of interest (e.g. ‘the cost of irreversibility’), these authors focus on the idea that stability could be provided by reaction kinetics, whenever there are autocatalytic or replication processes involved (i.e. what they refer to as ‘dynamic kinetic control’ [13–15]). This is indeed a relevant contribution to the field, coherent with the state-of-the-art experimental research, yet we feel that the problem, right from the beginning, presents more variables than just molecular kinetics (see below).

Another recent contribution that could hold some resemblance with ours is the ‘synergism hypothesis’ [71], since it defends that cooperative phenomena of various kinds may synchronize and produce novel, combined functional advantages, as a result of which ‘synergistic wholes’ would become the actual *units of evolutionary change*. However, even if this account of ‘function’ highlights it as a relational concept, as we do, the theoretical framework where it is embedded is very different, centred on the evolution of biological complexity. Corning and Szathmary develop their ideas from an evolutionary theory background, discarding the paradigms of ‘self-organization’ and classical ‘complexity sciences’ as significant contributions to the problem. In contrast, we propose a full integration between the two traditions. With that aim, a first approximation to *a theory of autonomous functional systems* is put forward in the next section, including ingredients from evolutionary biology and also from physics and chemistry, so that a better understanding about basic/minimal biological organization can be reached (i.e. about the actual units of life, not just about abstract ‘units of evolution’).

## 4. Outline of a theory of autonomous functional systems

**Figure 2.**
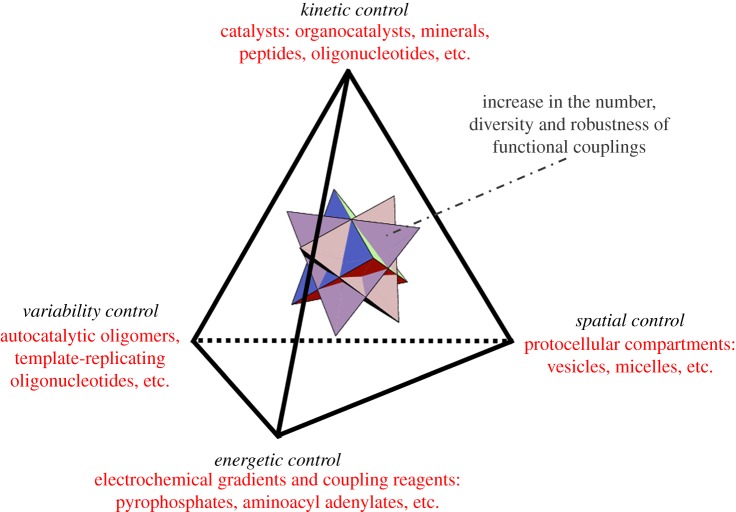
Schematic of the minimal set of control mechanisms that should operate, in a highly interdependent manner, to support the development of the first autonomous functional systems in the context of heterogeneous prebiotic chemistries. These mechanisms involve: (i) *kinetic control*, to coordinate the different reaction processes in time and enable transformations that reinforce the incipient chemistry but would otherwise be kinetically hindered; (ii) *spatial control*, to establish a clear inside/outside distinction and preserve minimal concentration thresholds of the relevant chemical species; (iii) *energetic control*, to facilitate the thermodynamically uphill but necessary reactions; and (iv) *variability control*, for these systems to have a minimal chance to evolve, through NS, into more complex forms. These four types of control mechanisms are represented as the vertices of a tetrahedron (with candidate molecular and supramolecular components suggested in red) and connected to each other by integrative and cooperative chemical couplings (black edges). Individuals with such an irreducible heterogeneity in their composition (and complexity in their dynamic behaviour) would constitute proper candidates as *units of selection* in this extended framework to conceive prebiotic chemical evolution. As a result of that evolutionary process, one could envision further functional diversification and more efficient integration (through new coupling processes), which would confer higher dynamic robustness to increasingly complex individuals in the population (a stage that is schematically illustrated by the multiple tetrahedron within the initial, elementary one).

It is out of the scope of this short critical review to give a full account of the emergence of autonomous (bio)chemical functions. In fact, the empirical results and theoretical insights delivered so far by the field of systems chemistry are not yet fully ripe to tackle such a task. Nevertheless, we would like to put on the table a concrete hypothesis, with the aim to illustrate the global idea that the problem involves several irreducible dimensions and, therefore, must be given a multidimensional solution. This is depicted in figure 2, which shows four different though interdependent types of *control mechanisms* that, according to our view, are fundamental requirements to assemble autonomous functional systems with evolutionary potential to become biological.

The implementation of those mechanisms could be achieved by different molecular components, but our thesis is that all of them: (i) must be produced from a common web of interlinked reaction and transformation processes; and (ii) must play a role in the maintenance of such a web, that is, they become intrinsically *functional*, as distinguishable parts of a system with differentiated contributions to its maintenance. Technically speaking, they are *constraints* on the dynamic behaviour of other material components of the system, and they exert mutual reinforcing influences on each other (i.e. they constitute *functionally coupled* constraints). The autonomy of the system therefore comes from its capacity to synthesize those components that, precisely, turn functional [72,73]. In other words, not all components of the system need to be internally synthesized (i.e. many will actually be taken up from the environment) but those that become functional should derive from—or be directly involved in— the incipient, protometabolic reaction network.

This is why it is so important, in the design and implementation of experiments, to work with molecular precursors rather than with final functional products—such as biopolymers or their aggregates. And this is why the problem of origins of life is, ultimately, a *chemical* problem. Nevertheless, we have no chance to find the relevant chemistry towards life unless we think about it from biology: from our knowledge of current living beings and, in particular, of the core functions performed by universal biochemical mechanisms. This takes us back to figure 2, and to the explanation of why we have chosen a tetrahedron as the geometry that best captures our proposal, which assumes that those functions (control mechanisms) can be implemented chemically, as is briefly described in the next paragraphs.

### 4.1. Kinetic control

This is the most evident control mechanism to be introduced, already considered pivotal by a majority of researchers. In fact, most origins-of-life experts conceive the problem as a transition from thermodynamic to kinetic control, eventually championed by genetically encoded enzymes [74,75]. So, the main difficulty around this vertex of the tetrahedron is not highlighting its importance, but finding what type of components could have carried out catalytic tasks before the advent of stereospecific protein enzymes. The field of organocatalysis should provide many potential candidates, but the methods applied in this area of research are typically looking for the optimization of single synthetic processes, mostly for the chemical industry, and they lack a prebiotic, systems view in their approach. Oligopeptides constitute the most parsimonious option, without excluding oligonucleotides that could have also played catalytic roles, like ribozymes do in current biochemistry. However, a lot has to be explored in this direction, including the contribution of mineral surfaces or multi-layers, metal ions or other types of low-molecular-weight compounds that could work as co-factors. Also, the presence of soft supramolecular structures (e.g. fatty acid- or lipid-made vesicles) may have an effect on the yield and the kinetics of processes that are not so favourable in bulk aqueous solution [76,77]. This brings us to the right-hand vertex of the tetrahedron depicted in figure 2.

### 4.2. Spatial control

Together with the temporal coordination of reaction processes, it is fundamental to develop mechanisms that harness the movement and spatial distribution of the various molecules that constitute the system. This is required not only to avoid dilution effects in free solution, which are always a threat to its cohesiveness, but also to enrich chemical behaviour itself—like Turing demonstrated long ago [78] and other authors have also highlighted as a key for the problem of life origins [79,80]. Indeed, the concentrations and spatial organization of the molecular components of all living beings (and, thereby, their metabolic activity, robustness and viability under changes in the external conditions) depend critically on their cellular nature. Furthermore, the actual identity and individuality of living systems is defined through the synthesis of their own boundaries and the mechanisms of interaction with their environment, which are usually anchored to the cell membrane [72]. Extensive experimental work during the last decades suggests that, at a very primitive stage of prebiotic chemical evolution, these boundaries were probably lipidic in nature, yet involving much simpler molecular structures and compositions than current ones [81–83].

### 4.3. Energetic control

In addition to kinetic and spatial control mechanisms, energy transduction is also at the heart of the problem, precisely because some of the necessary synthetic processes tend to be endergonic, and thus need to be coupled to exergonic ones. In a prebiotic context, these thermodynamic hurdles could be overcome through the activation of substrates with coupling reagents (e.g. cyanamide, carbodiimides and carbonyldiimidazole) [84–87] or making use of electrochemical gradients established across lipidic membranes [88]. In addition, pyrophosphate may have played the role of a primitive energy currency, while aminoacyl adenylates would represent an activated form of amino acids with a potential evolutionary connection with central molecules of the current translational machinery [89,90].

Thus, kinetic, spatial and energetic control mechanisms must interweave to bring about chemistries that support the minimum levels of complexity required for a system to become functionally autonomous [72]. In this context, cooperative events that make a new catalyst and/or a new energetic currency accessible, facilitating the synthesis of other components of the network, can be promoted (even if they imply a transition to higher levels of chemical complexity) if they confer a *selectable advantage* to the system as a whole, increasing its overall robustness. At some point, though, a ‘trade-off’ between complexity and stability will show up: the higher the complexity of the functional components and their relationships, the more difficult it becomes retaining them, without changes, in the system. After all, self-maintenance is based on far-from-equilibrium reaction cycles in which there is a continuous turnover of the molecular species involved. This reveals an unavoidable evolutionary bottleneck in any realistic prebiotic scenario, and this is why the fourth vertex of the tetrahedron, related to the capacity of controlling variability, must be included.

### 4.4. Variability control

It seems reasonable to assume that, at first, there would be little control on variation in these compartmentalized chemical mixtures. But, as we said above, the efficacy of NS depends on the appearance of mechanisms through which an accurate transmission of key features of these systems to their ‘progeny’ should be guaranteed—or, at least, turn statistically significant. Further research, including experimental work, must be carried out in order to determine how prominent variability control turns out to be during the initial stages of the evolutionary process, when system reproduction is probably a rather stochastic event [66,91,92]. Yet, there is no doubt that it must come into play, along with the implementation of mechanisms of heredity showing increasing reliability, for these prebiotic systems to overcome the abovementioned complexity–stability bottleneck.

In relation to this point, the capacity for cyclic and stationary reproduction regimes could well come about only as a result of subsequent stages in which a stronger degree of functional integration is achieved (see the mid-upper part of figure 1, roughly corresponding to the inner polyhedron in figure 2). Nevertheless, those stages could not develop out of the blue: the previous co-localization and coordination of supramolecular assemblies (e.g. vesicles and coacervates), catalysts (e.g. small-molecule organocatalysts, peptides or short oligonucleotides) and some energy currencies (e.g. electrochemical gradients and coupling agents, like pyrophosphate or some other ATP precursor) would be required [29]. The emergence of complex chemistries that resemble biochemistries (represented in the upper layers of figure 1) therefore implies, according to our view, the previous synthesis of a relatively wide variety of relatively simple molecular species and supramolecular assemblies (in the lower layers of figure 1), which have immediate effects on those actual synthetic processes, reinforcing their couplings and overall dynamic stability.

## 5. Implications for the origins of life

The most important—and most difficult—aspect of the problem, as it has been approached here, is to determine specific sets of processes and chemical couplings that could support the coordination of the primary functions required to put together and maintain prebiotic autonomous systems. According to what we just emphasized at the end of the previous section, the minimal functional systems that we are focusing on should be generally considered as *protocells*, as they involve heterogeneous assemblies of molecules in compartments whose dynamic behaviour is intrinsically linked with chemical reactions and various other processes [93].

Chemistry, fortunately, offers multiple options for the emergence and development of those composite protocellular individuals: (i) direct reaction couplings (i.e. transformation processes that share one or more chemical species); (ii) negative and positive feedback loops (autocatalytic cycles); (iii) stoichiometric couplings between several autocatalytic cycles, *a la Ganti* [94,95]; (iv) physical restrictions on molecular movement (by means of diverse kinds of boundaries); (v) osmotic couplings, if the chemical system is encapsulated within a volume-changing compartment (like a lipid vesicle) [96]; (vi) self-assembly processes (sustaining supramolecular structures); (vii) oligomerization reactions; (viii) endergonic–exergonic couplings (like (i), but with an energy currency involved); (ix) catalyst-mediated couplings (like in reflexive autocatalytic sets, *a la Kauffman*) [53,55]; and (x) regulatory couplings (second-order control mechanisms, in response to internal/external perturbations) [97].

These options are not mutually exclusive: they could operate in parallel, or intermingled. Several components from the vertices of the tetrahedron in figure 2, or combinations thereby (e.g. a peptide inserted into a lipid membrane), could actually be involved in different kinds of those couplings. The main point here is that a relatively simple (pre-biopolymer) chemical scenario already comprises plenty of diversity, both in terms of material components and processes of transformation. In fact, the degree of diversity is such that it is bound to trigger a combinatorial explosion of variables and parameters, difficult to handle. The new methodologies that systems chemists are developing to analyse complex molecular mixtures and heterogeneous assemblies will be helpful in this regard [28,98,99]. However, the problem of combinatorial explosion will not be tamed unless we integrate knowledge from synthetic biology and the top-down approaches toward achieving minimal cells in the laboratory [100,101], in order to restrict chemical options and to design experiments involving the proper suite of molecular precursors under prebiotically relevant conditions.

Furthermore, the required experimental work should be done not only under constant environmental conditions, but also in competitive scenarios, i.e. in interaction with other protocellular systems with similar energetic and material requirements [66,68,92]. If resources (i.e. substrates or energy supplies) are kept limited, this competition will eventually lead to selective dynamics in which certain combinations of functions (e.g. those who prove to be more efficient, more robust, etc.) will remain in the population. Therefore, a correlation between functional properties, coupling and integrative mechanisms, NS and evolutionary dynamics should be drawn.

**Figure 3.**
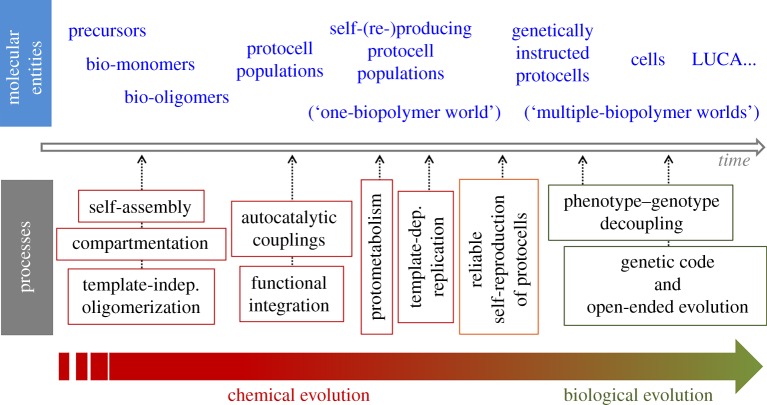
Scheme showing the main evolutionary transitions proposed for prebiotic chemistry and the overall process of origins of life. The notion of protocell populations employed here is remarkably wide, understood as heterogeneous, compartmented and functionally integrated *assemblies* of molecules that could implement, in successive stages, the different processes, capacities and mechanisms shown at the lower part of the figure.

In this respect, our intuition is that during a first period of prebiotic chemical evolution, *fitness* would be simply associated with the dynamic stability of protocellular systems, taken as individual units. Then, as a result of the collective dynamics and interactions that are bound to occur in this context (not only competitive, but also including collaborative or ‘symbiotic’ interactions), individuals with a more complex and functionally integrated organization would possibly come about (again, represented by the inner polyhedron in figure 2). The new properties of these selected individuals (e.g. more reliable reproduction, higher control of variability and more efficient catalysis), would, in turn, allow for new evolutionary dynamics, with a stronger operational power of NS. This would get progressively closer to an open-ended evolution type of process, like the one we observe in the current biosphere (corresponding to the upper layer of figure 1). The key evolutionary transitions that could have occurred in the proposed scenario, together with the corresponding molecular entities involved, are depicted in figure 3. The later stages of prebiotic evolution (sketched on the right-hand side of figure 3) are also relevant, but lie outside the scope of the present contribution.

## 6. Conclusion

In this prospective critical review, we have focused on the first steps of the process of the origins of life, which have important implications for subsequent stages. Ours constitute a non-conventional approach to prebiotic evolution, because it shifts the attention from homogeneous populations of molecules to populations of heterogeneous, compartmentalized and functionally integrated assemblies of molecules. The consequences of such a shift of perspective are multiple, both at the level of the individual units—which require an adequate characterization as self-maintaining systems with an internal organization—and also in terms of their collective and long-term evolutionary dynamics, based on competition, collaboration and selection mechanisms that are in need of further investigation.

The fact that such compartmentalized individuals possess an internal organization allows speaking about *function* in a physiologically relevant sense, because one can distinguish between parts of the system that contribute in a distinctive way to its maintenance as a whole. Immediate research goals to be targeted, in this context, will be: (i) the implementation of feasible versions of these composite systems, under specific experimental conditions; and (ii) the careful analysis and characterization of the roles played by the various kinds of molecules involved in the integrative process, ascribing functional properties to each of them. In this way, the concept of function has good chances to get naturalized, opening a scientific research programme to discover its deep chemical roots. As a result, new perspectives and theoretical approaches to understand evolvability as a general property of matter, well-grounded in experimental data, should also be brought forth.
